# Resistant *Streptococcus pneumoniae* strains in children with acute otitis media– high risk of persistent colonization after treatment

**DOI:** 10.1186/s12879-018-3398-9

**Published:** 2018-09-25

**Authors:** Izabela Korona-Glowniak, Piotr Zychowski, Radoslaw Siwiec, Elżbieta Mazur, Grażyna Niedzielska, Anna Malm

**Affiliations:** 1 Department of Pharmaceutical Microbiology with Laboratory for Microbiological Diagnostics, Medical University of Lublin, Chodzki 1 Street, 20-093 Lublin, Poland; 2 Department of Pediatric Otolaryngology, Phoniatrics and Audiology, Medical University of Lublin, Lublin, Poland; 3 Department of Medical Microbiology, Medical University of Lublin, Lublin, Poland

**Keywords:** Acute otitis media, *Streptococcus pneumoniae*, Risk factors, Antibiotic resistance, MLST

## Abstract

**Background:**

Despite advances in the development of pneumococcal conjugate vaccines, acute otitis media (AOM) is a common childhood infection, caused mainly by *Streptococcus pneumoniae*. It has been suggested that persistence of pneumococcal nasopharyngeal carriage is a risk factor for subsequent recurrent infections.

**Methods:**

In this study we evaluate the relationship between 55 pneumococcal strains obtained from nasopharynx/oropharynx (NP/OP) and middle ear fluid (MEF) of 62 children, aged between 1 and 16 years, during AOM (including recurrent/treatment failure AOM, and post-treatment visits), based on their phenotypic and genotypic characteristics performed by analyses of serotype, antibiotic susceptibility patterns and multilocus sequence typing.

**Results:**

*S.pneumoniae* was isolated from 27.4% of MEF samples; it constituted 43.6% of all positive bacterial samples from MEF samples. There was statistically significant concordance between isolation from the MEF sample and NP/OP colonization by *S. pneumoniae* (*p* < 0.0001). During post-treatment visits *S.pneumoniae* was isolated from 20.8% of children; 91% of them were positive in pneumococcal NP/OP culture during AOM. The serotypes belonging to 10- and 13-valent pneumococcal conjugated vaccines constituted 84% and 92% of the strains, respectively. Multidrug resistance was found in 84% of the strains. According to multivariate analysis, pneumococcal colonization after antibiotic therapy was significantly associated with shorter length of therapy in children with bilateral AOM.

**Conclusions:**

High persistent prevalence of antibiotic-resistant *S.pneumoniae* strains in children with AOM after unsuccessful bacterial eradication may presumably be regarded as a predisposing factor of infection recurrence.

**Electronic supplementary material:**

The online version of this article (10.1186/s12879-018-3398-9) contains supplementary material, which is available to authorized users.

## Background

*Streptococcus pneumoniae* is both a commensal bacterium colonizing the human nasopharynx asymptomatically and a pathogen responsible for a wide spectrum of diseases, ranging from mild respiratory tract infections to severe invasive diseases. It is a major cause of morbidity in children and can be isolated in 25–60% of nasopharyngeal samples obtained from healthy children. Nasopharyngeal colonization can lead to infection, by spreading to adjacent mucosal tissue to cause acute otitis media (AOM) or pneumonia, or by blood stream to other sites causing bacteriemia, meningitis or focal infections [1]. The worldwide increase in antibiotic resistance in *S. pneumoniae* has been related to the spread of several pneumococcal serotypes (6A, 6B, 9 V, 14, 15A, 19F, 19A, and 23F), the so-called ‘paediatric serotypes’ which mainly belong to a small number of pneumococcal clones which nomenclature is standardized by the Pneumococcal Molecular Epidemiology Network (PMEN) [2–5].

Considering advances in the development of pneumococcal conjugate vaccines (PCVs) leading to reduction of invasive disorders, pneumococcal diseases are still problem for public health. The impact of the first introduced pneumococcal conjugate vaccine (PCV7) containing seven capsular antigens of serotypes 4, 6B, 9 V, 14, 18C, 19F and 23F on the decrease of invasive pneumococcal disease has been significant in infants, older children and adults. A decrease in rates of antimicrobial resistance among pneumococcal isolates was observed as an additional benefit of the vaccine because resistance to penicillin, macrolides and multidrug resistance are mostly associated with serotypes included in PCV7, namely 6B, 9 V, 14, 19F and 23F [6]. In 2007, World Health Organization (WHO) recommended to introduce PCV to the national infant immunization programs of all countries. Lower proportion of pneumococcal infections caused by PCV7 serotypes has been noted only in countries with routine effective use of PCV7 [7].

Acute otitis media (AOM) is a common childhood infection, occurring most frequently as a consequence of viral upper respiratory tract infections, but treated mainly with antibiotics. The leading causes of bacterial AOM worldwide are *Streptococcus pneumoniae*, non-typeable *Haemophilus influenzae*, *Moraxella catarrhalis*, and group A *Streptococcus* [8–10]. In approximately 80% of children aged 2–5 years AOM is diagnosed at least once, and 30–40% of them have recurrent episodes [8, 11]. More than 10% of children do not improve despite antibiotic therapy. It was noted that history of previous recurrent episodes of AOM, selective pressure of previous antibiotic courses and age below 2 years are risk factors related to this entity [12]. *S. pneumoniae* was shown to be the most prevalent pathogen among AOM patients who had failed a course of antibiotic therapy [12, 13]. It was demonstrated that the persistence of pneumococcal nasopharyngeal carriage is a risk factor for subsequent recurrent infections and the antimicrobial selection should have an impact on nasopharyngeal colonization [14, 15]. The nasopharynx is often a reservoir of bacteria involved in AOM where they interact with each other and the host’s immune system [16]. Disturbance of existing balance in this microbiota may facilitate the colonizing bacteria to expand through the Eustachian tube into the middle ear and cause infection [17].

In our previous study, we observed the high prevalence of antibiotic-resistant otopathogens in recalcitrant AOM and the high colonization rate by the same otopathogen species after completion of antibiotic therapy [10]. In this study we evaluate the relationship between the pneumococcal strains obtained from the nasopharynx (NP) and oropharynx (OP) and the middle ear fluid (MEF) of children during AOM and during post-treatment visits, based on their phenotypic and genotypic characteristics performed by analyses of serotype, antibiotic susceptibility patterns and multilocus sequence typing (MLST).

## Methods

### Patients

This prospective study enrolled 62 children, aged between 1 and 16 years who were diagnosed with AOM by an Ear-Nose-and-Throat (ENT) specialist during 2010–2014 in the Department of Pediatric Otolaryngology, Phoniatrics and Audiology, Medical University of Lublin, Poland. From all children’s parents, written informed consent was obtained. The Ethical Committee of the Medical University of Lublin approved the study protocol (No. KE-0254/75/211). Patients were referred to tympanocentesis according to criteria proposed by Bluestone [18] and extended with recurrent AOM cases. Only a small percentage of patients fulfills the criteria for the tympanocentesis: very severe pain (none in our study), symptoms of toxemia, unsatisfactory reaction to antibiotic therapy (treatment failure), developing complications of AOM, AOM in neonatal and immunosupressed patients (none in our study). All tympanocentesis were performed as an emergency treatment. Patients with recurrent AOM were included only if the microbiological material was taken during active acute inflammatory state, not planned tympanic membrane tube insertion.

A case of AOM was defined when a patient had acute onset of signs lasting 7 or more days with symptoms including fever, otalgia and/or irritability, with presence of middle ear fluid (MEF) and with signs of middle-ear inflammation detected by videootoscopic examination (e.g tympanic membrane erythema and bulging). Recurrent AOM was defined as a history of 3 (or more) episodes in the previous 6 months or 4 (or more) episodes in the previous 12 months; AOM treatment failure defined by persistence of AOM signs and symptoms after at least 48 h of antibiotic therapy or the recurrence of AOM signs and symptoms within 30 days of completing a course of antibiotics; a new case was defined as the appearance of AOM signs and symptoms after a 30-day symptom-free period after completing a course of antibiotics. A total of 16 (25.8%) children received an ongoing oral antibiotic therapy at the admission to hospital. Children were immunized by a antipneumococcal and/or anti-*Haemophilus influenzae* type b vaccines in 21% and 93.5%, respectively. Demographic and selected clinical data of studied children are shown in Table 1.

**Table 1:** Demographic and clinical characteristics of children with AOM

Characteristics	Category	Total (%) *n* = 62	No of SP positive samples (%)		
			MEF during AOM	NP/OP during AOM	NP/OP during PT visit^c^
Age (yr)	1–2	20 (32.3)	6 (30.0)	5 (25.0)	5 (25.0)
	3–5	31 (50.0)	10 (32.3)	11 (35.5)	5 (21.7)
	≥6	11 (17.7)	1 (9.1)	1 (9.1)	1 (10.0)
Sex	Male	38 (61.3)	13 (34.2)	12 (31.6)	8 (24.2)
	Female	24 (38.7)	4 (16.7)	5 (20.8)	3 (15.0)
Sibling possessing	No	21 (33.9)	6 (28.6)	5 (23.8)	4 (23.5)
	1	30 (48.4)	11 (36.7)	11 (36.7)	7 (25.9)
	≥2	11 (17.7)	0 (0.0)	1 (9.1)	0 (0.0)
DCC/school attendance		45 (72.6)	12 (26.7)	12 (26.7)	7 (18.9)
Laterality	Unilateral	25 (40.3)	5 (20.0)	5 (20.0)	2 (9.1)
	Bilateral	37 (59.7)	12 (32.4)	12 (32.4)	9 (29.0)
Category of AOM	TF	37 (59.7)	10 (27.0)	11 (29.7)	7 (24.1)
	R	6 (9.7)	3 (50.0)	1 (16.7)	1 (16.7)
	N	19 (30.7)	4 (21.1)	5 (26.3)	3 (16.7)
CRP (mg/L) (median, range)^a^		2.27 (0.01–46.9)	3.6 (0.15–12.0)	4.8 (0.05–19.5)	NA
WBC (G/L) (median, range)^b^		14.0 (5.4–33.0)	20.4 (7.8–30.2)	17.6 (7.8–30.2)	NA
Ongoing antibiotic therapy at the admission to hospital	No	46 (74.2)	15 (32.6)	15 (32.6)	10 (24.4)
	Yes	16 (25.8)	2 (12.5)	2 (12.5)	1 (8.3)
Antibiotic therapy at the hospital	AM/AMC	11 (17.7)	3 (27.3)	1 (9.1)	1 (11.1)
	CXM	21 (33.9)	4 (19.1)	5 (23.8)	4 (20.0)
	CTX/CAZ	28 (45.2)	10 (35.7)	11 (39.3)	6 (26.1)
	CLI	2 (3.2)	0 (0.0)	0 (0.0)	0 (0.0)
Antipneumococcal vaccination		13 (21.0)	1 (7.7)	0 (0.0)	0 (0.0)
AntiHib vaccination		58 (93.5)	17 (29.3)	17 (29.3)	11 (22.5)

^a^data available for 50 persons; ^b^data available for 61 persons; ^c^data available for 53 persons; *R* recurrent AOM, *TF* treatment failure AOM, *N* new case AOM, *MEF* middle ear fluid, *DCC* day care center, *CRP* C-reactive protein, *WBC* white blood cells, *PT* visit post-treatment visit, *Hib Haemophilus influenzae* type b, *NP* nasopharyngeal, *OP* oropharyngeal, *AM/AMC* amoxicillin or amoxicillin-clavulanic acid, *CXM* cefuroxime, *CTX/CAZ* cefotaxime or ceftazidime, *CLI* clindamicin, *NA* not applicable

### Study procedures

**Fig. 1:**
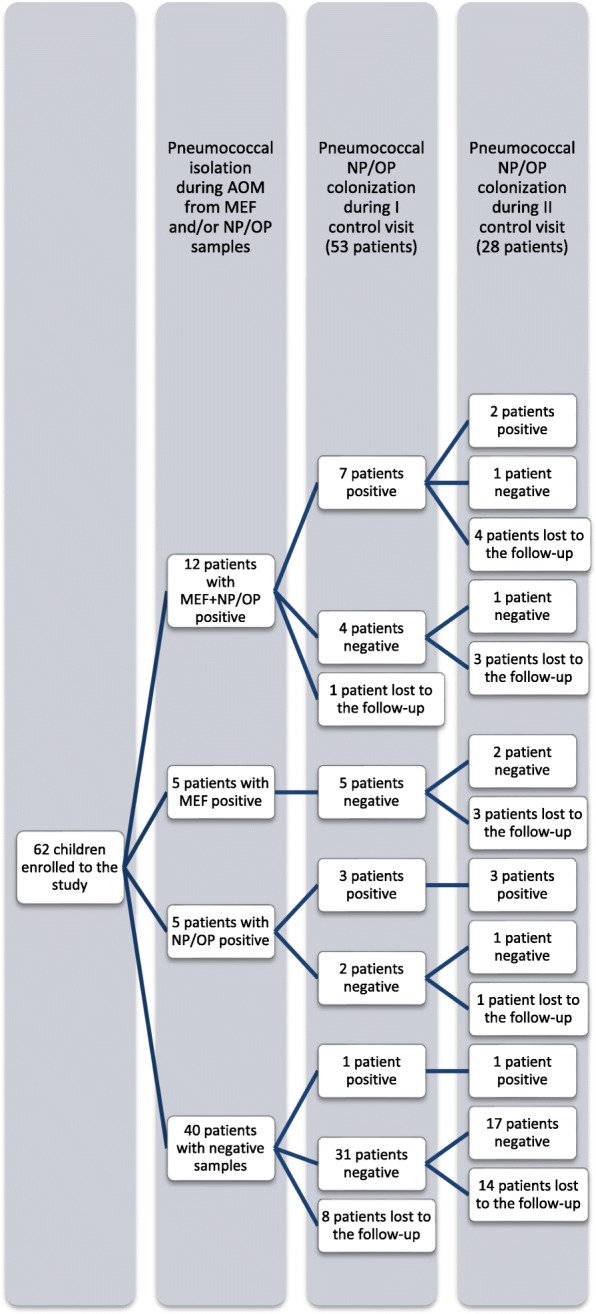
Flow chart for pneumococcal isolation/colonization analysis. (Middle ear fluid (MEF) specimens, nasopharyngeal and oropharyngeal (NP/OP) swabs were collected during AOM visits. Two post-treatment visits were carried out: after the antibiotic therapy (post-treatment visit I) and two weeks after the antibiotic therapy (post-treatment visit II). NP and OP swabs were collected during these visits. Of all patients 53 patients participated in post-treatment visit I and 28 patients in both visits. A total of 348 microbiological cultures were analyzed)

NP and OP swabs, and MEF specimens were collected at the same time during AOM from 62 children. Next, first-line or second-line antibiotic treatment was administered for the average period of 9 days (median 9, range 4–23, mean 9.6, Standard Deviation (SD) ±3.2) with the use of amoxicillin or amoxicillin-clavulanic acid, cefuroxime, cefotaxime or ceftazidime and clindamicin (Table 1). Two post-treatment visits were carried out: after the antibiotic therapy (post-treatment visit I) and two weeks after the first visit (post-treatment visit II). All post-treatment visits were performed by ENT specialist (the same for all patients). Medical interview, videotoscopic evaluation of tympanic membrane, tympanometry and hearing level examinations (otoacoustic emisions, pure tone audiometry – depending on patients age and cooperation) were provided during these visits as well as collection of both NP and OP swabs. 28 patients participated in both visits and 53 patients only in post-treatment visit I. A total of 382 microbiological cultures were analyzed (Fig. 1). Due to small number of patients in the post-treatment visit II only the data from post-treatment visit I were included to further statistical analysis.

The nasopharyngeal (NP) and oropharyngeal (OP) specimens were obtained with the sterile alginate-tipped swabs on aluminium or plastic shafts, respectively. Antisepsis of the ear canal before tympanocentesis was performed by installing drain soaked with Octenisept (Schülke & Mayr) for 1 min. After removing it by suction, myringotomy was carried out, during which dense, mucopurulent discharge draining under pressure from middle ear was found uni- or bilaterally. MEF samples were collected from the ear with the use of sterile suction needle inserted into the middle ear cavity through the incision in the tympanic membrane. The samples were placed in sterile eppendorf tubes maintained in 37 °C, and transported immediately to the laboratory. The remaining amount of exudate had been aspirated. Next, in 29 (46.8%) patients tympanostomy tube was placed.

### Laboratory procedures

Swabs and MEF samples were inoculated on selective Mueller-Hinton agar with 5% sheep blood and 5 mg/L of gentamicin for selective cultivation of streptococci. The identification and serotyping of *S. pneumoniae*, and susceptibility testing to selected antibiotics were performed in the same manner as in our previous researches [19, 20].

### Multilocus sequence typing (MLST) analysis

Bacterial genomic DNA of all 25 isolates was prepared with Genomic Mini Kit (A&A Biotechnology, Gdynia, Poland) and used as templates for PCR. MLST analyses were performed as described in our previous study [19].

### Statistical analysis

Data processing and analysis were performed using Tibco Statistica Ver. 13.3 (TibcoSoft. Inc.). The results are expressed as percentage or median with range. Univariate analyses were performed using Chi-squared or Fisher exact test, depending on size of samples and of contingency tables for categorical variables and using Mann-Whitney U test for continuous variables. Odds ratios (OR) and their 95% confidence intervals (CI) were calculated. Logistic regression models were fitted to identify risk factors associated with *S. pneumoniae* AOM cases comparing to other ones. From these models, adjusted odds ratios (OR) and 95% Confidence Intervals were derived; corresponding *p*-values were those from Wald’s test. Goodness of fit was checked using Hosmer and Lemeshow’s test. Statistical significance was set if the 2-tailed *p* value was < 0.05.

## Results

### The prevalence and affecting factors of *S. pneumoniae* isolations from MEF and NP/OP samples during AOM and post-treatment visits

*S. pneumoniae* was isolated as a single otopathogen from 22.6% of MEF cultures and from 4.8% of MEF samples in polymicrobial cultures. *S. pneumoniae* was isolated from MEF cultures of 17 (27.4%) patients and from NP and/or OP (NP/OP) swabs of 17 (27.4%) patients during AOM. Simultaneous pneumococcal isolation in MEF and NP/OP samples in 12 (19.4%) patients (70.6% of *S. pneumoniae* positive MEF samples) revealed statistically significant concordance between isolation from the MEF sample and NP/OP colonization by *S. pneumoniae* during AOM (*p* < 0.0001).

During the post-treatment visits pneumococcus was isolated from 11 (20.8%) children; 10 of them were positive in pneumococcal culture during AOM. In one patient, *S. pneumoniae* isolates appeared during the post-treatment visit for the first time (Fig. 1).

Even though the percentage of pneumococcal isolations was higher from children with treatment failure and recurrent AOM (in MEF sample (31%) and NP/OP colonization during post-treatment visit (22.9%) than from new case AOM (21.1% and 16.7%, respectively), there was no significant difference in the isolations of *S. pneumoniae* relating to different clinical manifestation of AOM. Frequency of NP/OP colonization by pneumococci during AOM was nearly equal for patients with treatment failure or recurrent AOM and new case AOM (27.9% and 26.3%, respectively) (Table 1).

According to multivariate analysis, ongoing antibiotic treatment at the admission to hospital was negatively associated risk factor, whereas WBC and CRP values were independent factors positively associated with *S. pneumoniae* as a otopathogen isolated from MEF during AOM (Table 2). Bilateral AOM and shorter length of antibiotic therapy (median 9.0, range 4–12 days, mean 8.2, SD ± 2.5 in colonized patients vs. median 9.5, range 6–23 days, mean 9.9, SD ± 3.1 in non-colonized patients) were risk factors associated with pneumococcal colonization after treatment (Table 3).

**Table 2:** Associations of epidemiological factors to prevalence of *Streptococcus pneumoniae* in middle ear fluid in children with AOM - univariate and multivariate analysis

Characteristics	Univariate analysis		Multivariate model	
	OR (95% CI)	*P* value	OR (95% CI)	*P* value
Age (yr)	0.8 (0.61–1.06)	0.13		
Male sex	1.6 (0.86–3.03)	0.14		
Sibling possessing	0.96 (0.53–1.72)	0.88		
DCC/school attendance	0.93 (0.50–1.73)	0.83		
Bilaterality	1.38 (0.76–2.52)	0.29		
Category of AOM:TF/R	1.28 (0.67–2.42)	0.46	0.05 (0.01–0.17)	0.062
CRP (mg/L)	1.05 (0.97–1.14)	0.23	1.12 (1.01–1.25)	0.039*
WBC (G/L)	1.21 (1.07–1.36)	0.002*	1.22 (1.05–1.42)	0.009*
Ongoing antibiotic therapy at the admission to hospital	0.54 (0.24–1.21)	0.14	0.07 (0.006–0.87)	0.038*
Antipneumococcal vaccination	0.42 (0.14–1.2)	0.10	0.059 (0.002–1.57)	0.091

*Statistic significance; *R* recurrent AOM, *TF* treatment failure AOM, *N* new case AOM, *DCC* day care center, *CRP* C-reactive protein, *WBC* white blood cells

**Table 3:** Associations of epidemiological factors to *Streptococcus pneumoniae* colonization in children with AOM after treatment - univariate and multivariate analysis

Characteristics	Univariate analysis		Multivariate model	
	OR (95% CI)	*P* value	OR (95% CI)	*P* value
Age (yr)	0.8 (0.57–1.12)	0.20		
Male sex	1.35 (0.65–2.8)	0.43		
Sibling possessing	0.89 (0.44–1.78)	0.73		
DCC/school attendance	0.84 (0.42–1.68)	0.62		
Bilaterality	2.02 (0.9–4.6)	0.094	3.8 (1.1–13.0)	0.037*
Category of AOM:TF/R	1.21 (0.58–2.54)	0.60		
CRP (mg/L)	0.95 (0.82–1.09)	0.43		
WBC (G/L)	0.98 (0.87–1.1)	0.72		
Ongoing antibiotic therapy at the admission to hospital	0. 53 (0.18–1.57)	0.25		
Antibiotic therapy at the hospital	1.4 (0.57–3.46)	0.46		
Length of antibiotic therapy at the hospital	0.73 (0.51–1.04)	0.08	0.8 (0.71–0.93)	0.003*

*Statistic significance; *R* recurrent AOM, *TF* treatment failure AOM, *N* new case AOM, *DCC* day care center, *CRP* C-reactive protein, *WBC* white blood cells

### Pneumococcal strains analysis – Antimicrobial susceptibility, serotype distribution and vaccines coverage, and molecular typing

A total of 55 pneumococcal isolates were recovered from MEF (24 isolates, both from one or two ears) and/or NP/OP (17 isolates) during AOM and 14 isolates were collected from NP/OP during the post-treatment visits. All of the pneumococcal isolates were identified by phenotyping (serotyping, antimicrobial susceptibility) and genotyping (MLST method) which revealed similarity in most of isolates obtained from different samples from the same patients. Finally, the presence of 25 different pneumococcal strains was confirmed. In terms of isolation time, during AOM and during post-treatment visits 23 and 12 pneumococcal strains were isolated, respectively.

In all patients but one, in whom more than one isolate of *S. pneumoniae* was obtained both over successive visits or from multiple sites, it was the same pneumococcal strain (Table 4). One patient was colonized by two different strains during I and II post-treatment visits (strains 48A and 48B). Out of 22 patients whose samples were pneumococcal positive during AOM, in 12 (54.5%) patients the same strain was isolated from MEF and NP/OP samples during AOM (2–3 isolates from the same patient).

**Table 4:** Phenotypic and genotypic characteristics of 25 pneumococcal strains isolated from children with AOM

No of strain	Serotype	Antibiotic resistance pattern	Site of isolation			Sequence type	Predicted founder ST/CC^a^	Related PMEN clone
			AOM		PT visit			
			MEF R/L	NP/OP	NP/OP			
1	14	PECcSxt	+/+	+/−	+/−	143	156/CC1	Spain 9 V-3 DLV
2	19F	PTeCSxt	−/+	+/−	−/−	423	15/CC3	England 14–9 SLV
3	14	PECcTeSxt	+/−	+/−	NA	156	156/CC1	Spain 9 V-3
5	14	PECcTeSxt	+/−	+/−	+/+	156	156/CC1	Spain 9 V-3
10	19F	PECcTeCSxt	−/+	+/+	−/−	87	88/CC50	
11	23F	PECcTeCSxt	+/+	+/−	+/+	2033	81/CC13	Spain 23F-1 SLV
14	19F	PECcTeCSxt	+/−	−/−	−/−	87	88/CC50	
22	22F	S	−/−	−/+	−/−	433	433/CC23	
27	18C	S	−/+	−/−	−/−	496	496/CC192	
28	23F	PECcTeSxt	+/+	+/+	+/−	81	81/CC13	Spain 23F-1
30	6B	PECcCSxt	+/+	−/−	−/−	135	473/CC8	
31	14	PECcSxt	+/+	−/−	−/−	143	156/CC1	
33	19A	PECcTeSxt	−/−	+/−	−/+	320	320CC2	
35A	19F	PECcSxt	−/+	−/−	+/−	320	320/CC2	
35B	19F	PECcSxt	−/−	+/−	−/−	9062	320/CC2	
41	14	E	+/−	+/−	−/−	9	15/CC3	England 14–9
42	15A	PECcTe	−/−	−/+	+/−	374	63/CC10	Sweden 15A-25 SLV
44	14	PECcTe	−/+	+/−	+/−	10,340	156/CC1	
48A	14	PECcSxt	−/−	−/−	+/−	10,342	88/CC50	
48B	14	PECcTeCSxt	−/−	−/−	+/−	15	15/CC3	England 14–9 SLV
49	14	PECcTeSxt	−/−	+/−	+/−	156	156/CC1	Spain 9 V-3
55	14	PECcSxt	−/+	+/−	+/−	15	15/CC3	England 14–9 SLV
56	6B	PECcTeCSxt	−/−	+/−	−/−	135	473/CC8	
60	3	S	+/+	+/−	−/−	505	180/CC16	
62	19F	TeCSxt	+/+	−/−	−/−	423	15/CC3	England 14–9 SLV

*MEF* middle ear fluid, right (R)/left (L) ear, nasopharyngeal(NP)/oropharyngeal(OP) samples, *PT* post-treatment, *P* penicillin; *E* erythromycin, *Cc* clindamycin, *Te* tetracycline, *C* chloramphenicol, *Sxt* co-trimoxazol, *S* sensitive to all tested antibiotics, *PMEN* Pneumococcal Molecular Epidemiology Network, ^a^Predicted founders were assigned by comparing tested strain collection MLST data with whole MLST database

**Fig. 2:**
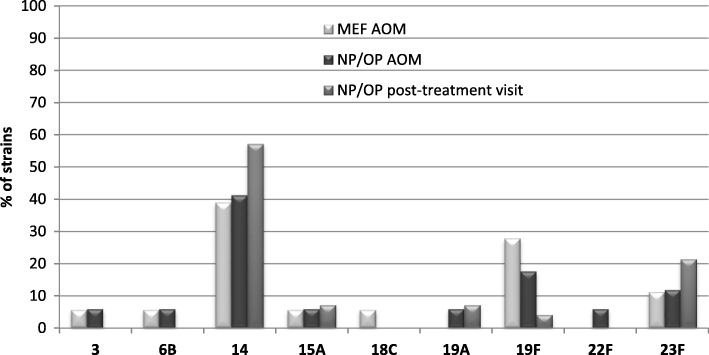
Serotype distribution for 55 pneumococcal isolates obtained during AOM and post-treatment visits. (AOM, acute otitis media; MEF, middle ear fluid; NP, nasopharyngeal; OP, oropharyngeal)

The most frequent among isolated strains were serotypes 14 (10/25 strains; 40%) and 19F (6/25 strains; 24%). Serotypes belonging to pneumococcal conjugated vaccines - PCV10 (containing serotypes 1, 4, 5, 6B, 7F, 9 V, 14, 18C, 19F, 23F) and PCV13 (containing serotypes 3, 6A, 19A additionally to 10-valent vaccine) constituted 84% and 92% of the strains, respectively. Some differences in frequency of serotypes distribution among strains isolated during AOM and during post-treatment visits were observed (Fig. 2).

**Fig. 3:**
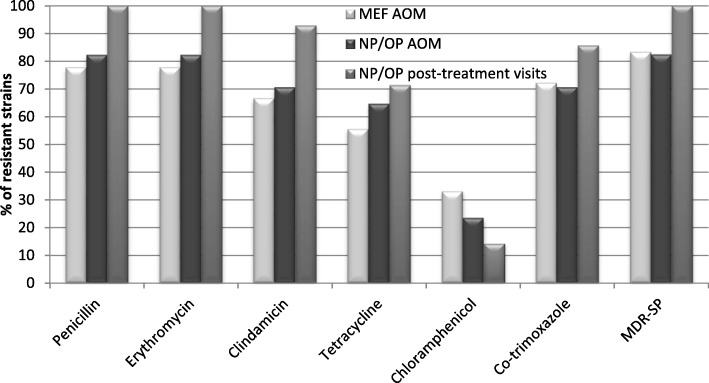
Resistance to antibiotics for 55 pneumococcal isolates obtained during AOM and post-treatment visits. (AOM, acute otitis media; MEF, middle ear fluid; NP, nasopharyngeal; OP, oropharyngeal; MDR-SP, multidrug resistant *S. pneumoniae*)

Susceptibility to all tested antimicrobial agents was observed in 12% (3/25 strains) of the pneumococcal isolates. These strains belonged to serotypes 3, 18C, 22F (1 isolate per each serotype). Among all of the strains, 80% showed decreased susceptibility to penicillin (PNSSP). Higher level of MIC values was shown for pneumococcal strains isolated during post-treatment visits of the patients after completion of the antibiotic therapy for all tested β-lactams (Table 5). Three strains had mutated during antibiotic treatment and two of them being intermediate became resistant to penicillin and one became resistant to cefotaxime. Eventually, there were differences during AOM and post-treatment visits in prevalence of intermediate and resistant pneumococcal isolates to penicillin (65.3% and 13% vs. 66.7% and 33.3%), to ampicillin (30.4% and 26.1% vs. 41.7% and 33.3%), to cefaclor (21.7% and 78.3% vs. 0% and 100%), and to cefotaxime (39.1% and 0% vs. 50% and 8.3%), respectively. 84% of *S. pneumoniae* strains were multidrug resistant (MDR-SP). Most of MDR-SP strains (90.5%) were non-susceptible to penicillin. Resistance analysis has revealed higher percentage of resistant strains isolated during post-treatment visits in comparison to strains isolated during AOM (Fig. 3). It was shown that among PNSSP, erythromycin- and clindamicin-resistant strains in 90% belonged to PCV10 serotypes and in 95% to PCV13 serotypes. When it comes to strains resistant to tetracycline, chloramphenicol and co-trimoxazole, coverage by PCV serotypes was from 86 to 100%.

**Table 5:** Minimal inhibitory concentrations (MIC) for selected β-lactam antibiotics for 55 pneumococcal isolates obtained during AOM and post-treatment visits

Antibiotics		Number of strains with MIC (mg/L)																		Range	MIC_50_	MIC_90_
		≤0.023	0.064	0.12	0.19	0.38	0.5	0.75	1.0	1.5	2	3	4	16	24	32	48	64	≥256			
Penicilin	MEF AOM	4			1	1	2	4	3	1	1									0.023–2.0	0.75	2.0
	NP/OP AOM	3		1	1		2	4	3	1	2									0.023–2.0	0.75	2.0
	PT visit				1	2	1		4		3	1								0.19–2.0	1.0	2.0
Ampicillin	MEF AOM	4			1	2		2		5	1	2								0.023–3.0	0.75	3.0
	NP/OP AOM	3		1	1	2		2		4	2	2								0.023–3.0	0.75	3.0
	PT visit			1		2		2	1	2	1	2	1							0.12–4.0	1.0	3.0
Cefaclor	MEF AOM					4		1			1			1	2	5	1	1	1	0.38–256	24	64
	NP/OP AOM					3	1			1	1			1	2	5	1		2	0.38–256	24	256
	PT visit									1		1		1	2	3	1	1	2	1.5–256	32	256
Cefotaxime	MEF AOM	4		1	2	1	3	4	1	1										0.023–1.5	0.5	1.0
	NP/OP AOM	3	1	1	2	1	3	3	1	1	1									0.023–2.0	0.5	1.5
	PT visit			1	1	1	2	4	2		1									0.12–2.0	0.75	1.0
Imipenem	MEF AOM	4	2	3	3	5														0.023–0.38	0.12	0.38
	NP/OP AOM	3	3	3	2	5		1												0.023–0.75	0.12	0.38
	PT visit		1	4	3	2	1	1												0.064–0.75	0.19	0.5

*MEF* middle ear fluid, nasopharyngeal (NP)/oropharyngeal (OP) samples, *PT* post-treatment

Among 17 sequence types (STs) identified, 2 STs were newly assigned - ST10340 and ST10342 (Table 4). The eBURST analysis by comparing tested strain collection MLST data with whole MLST database revealed that all of the STs were grouped into 10 clonal complexes (CCs). Four of the CCs containing 7 STs with 14 (56%) isolates belonged to the pandemic PMEN clones or their variants (SLV and DLV), included Spain9V-3-ST156 (6 isolates), England14–9-ST9 (5 isolates), Spain23F-1-ST81 (2 isolates) and Sweden15A-25-ST63-SVL (Additional file 1).

## Discussion

In this study of pneumococcal aetiology in children with different cases of AOM, high frequency of strains resistant to various antibiotic drugs was found. In fact, among all, 73.3% of isolated pneumococcal strains were obtained from patients with treatment failure and recurrent AOM. Antibiotics are prescribed at up to 80% of GP or ENT visits for AOM whereas antibiotic treatment is considered to be a selection factor of initially non-susceptible strains, facilitating colonization with new strains or development of resistance during therapy. *S. pneumoniae* was most prevalent pathogen isolated from MEF in patients who had failed a course of antibiotic therapy [13, 21]. Pichichero et al. [22] reported that 28% of AOM cases were caused by *S. pneumoniae* with high proportion of highly resistant strains and in 77% of cases isolation was done from recurrent AOM or initial treatment failure. Eldan et al. [23] found a significantly higher carriage rate of drug-resistant *S. pneumoniae* in patients with nonresponsive AOM than in children with simple, uncomplicated AOM who did not receive previous antibiotic therapy. In our study, 91.7% strains isolated from nasopharynx of patients with treatment failure and recurrent AOM were MDR ones. Complete concordance between resistance patterns and STs of pneumococcal isolates recovered from NP/OP and those isolated from MEF has proved that nasopharyngeal MDR strains were the etiologic agents of AOM in 70.6% (12/17) of patients with pneumococcal aetiology. Moreover, all of the pneumococcal isolates recovered from NP/OP during post-treatment visits after treatment were identical according to resistant patterns and STs with those isolated during AOM from the same patients. It is necessary to use molecular typing methods like MLST to find the genetic relatedness between bacterial isolates originating from middle ear cavity and naso/oropharynx. One study revealed with MLST analysis that 100% of pair pneumococcal isolates from nasopharyngeal and MEF samples were identical [24]. Using other genetic methods, it was observed that 71.4–80% of *S. pneumoniae* isolated from paired nasopharynx and MEF showed genetic identity [25, 26]. Our study and previous findings emphasize the hypothesis that nasopharynx acts as a reservoir for bacterial pathogens in AOM, including *S. pneumoniae*.

Tympanocentesis is not routinely performed procedure in Polish children which was the reason of relatively small number of enrolled patients. It is a limitation of this study. However, the results seem to confirm the relationship between the persistent pneumococcal colonization after unsuccessful eradication and presumably recurrence of an infection. It is worth noting that in 5 patients, whose MEF samples were *S. pneumoniae* positive during AOM, pneumococcal eradication was observed during post-treatment visit.

Some studies suggest that signs and symptoms of AOM caused by *S. pneumoniae* may be more severe (fever, severe earache, bulging TM) than those caused by other pathogens [27–29]. As well as it was observed elsewhere [30], in our study, higher values of CRP and WBC, whose levels rise in response to inflammation, were significantly important factors involved presence of *S. pneumoniae* in MEF sample in children with AOM.

A high level of penicillin-resistant *S. pneumoniae* is considered today the leading cause of antibiotic therapeutic failures in AOM [13–15, 23]. High frequency of PNSSP isolations in children with recalcitrant AOM, observed in our study, was also reported by other authors in other countries, including Poland [13, 23, 31, 32]. Furthermore, we noticed the difference between frequency of PNSSP isolations during AOM (86.4%, 19/22 strains) and during post-treatment visits (100%, 12/12 strains) caused by the selection pressure of used antibiotics. Moreover, in case of three strains isolated during post-treatment visits the increase of penicillin and cefotaxime MIC values were observed in comparison to isolates obtained during AOM.

Very high frequency (84%) of MDR strains in our studies has exceeded the data presented in other studies on AOM treatment failure, that 40–67% of pneumococcal strains were MDR [32, 33]. Our study have confirmed recent reports showing that antibiotic therapy in AOM induces selection of preexisting nasopharyngeal antibiotic-resistant *S. pneumoniae* [14, 34]. In our study, it was shown that too short time of therapy in children with bilateral AOM, but not of the type of β-lactams has the impact on persistent pneumococcal colonization after treatment.

In this study, the frequency of *S pneumoniae* isolation from MEF samples was 27.4% and it constituted 43.6% of positive MEF samples. There are similarities between our study and other studies using conventional culture method which reported relatively high rates of pneumococcal MEF isolations from children with recurrent AOM and AOM treatment failure [32, 35]. Presumably, in our study, this situation was directly related to antipneumococcal vaccination pursued only in 21% of tested children. There was a significantly lower frequency of pneumococcal colonization (0%) in immunized children both at the AOM (*p* = 0.015) and during post-treatment visits (*p* = 0.047). However, in one immunized patient *S. pneumoniae* isolate with serotype 6B was detected in MEF sample. A number of studies have examined the impact of antipneumococcal immunization with the use of conjugate vaccines on the burden of AOM. The reduction of visits for AOM, antibiotic prescription and tympanosomy tube placements were noted in these studies [13, 36]. However, in the Belgian study of children with the history of recurrent AOM immunized with PCV7 followed by a 23-valent polysaccharide booster, the authors reported no reduction in the number of AOM episodes and no changes in nasopharyngeal pneumococcal carriage during the 26-month follow-up period suggesting that the vaccine may not be useful when recurrent AOM is established [37].

After eight years since PCV7 introduction in United States it was reported that PCV7 serotypes virtually disappeared from MEF of vaccinated children with AOM [38]. High rate (73.7–80.1%) of PCVs serotype coverage of isolates colonizing upper respiratory tract in pre-school healthy children as well as in children with recurrent upper respiratory tract infections from south-east Poland was observed [20, 39]. According to Skoczynska et al. study from Poland [40], in children aged less than 5 years, the PCV10, and PCV13 covered 54.8%, and 68.8% of all IPD cases, and 76.3%, and 86.3% of cases involving children under 5 years of age. Our data showed that the *S. pneumoniae* serotype coverage by the currently available PCVs among isolates colonizing nasopharynx as well as otopathogens from MEF cultures in children with AOM from Poland is still very high (84–92%) and exceeded these reported in other European countries [35, 41]. A routine antipneumococcal vaccination has only been implemented in Poland in 2017 (https://gis.gov.pl/en/homepage/). Two years before, the PCVs had been recommended for children under 5 years old however with reimbursement only for sselected risk groups. National Institute of Public Health of National Institute of Hygiene annual data revealed slightly increasing percentage (from 1.5 to 4%) of vaccinated children aged 0–14 years in 2007–2014 period (http://wwwold.pzh.gov.pl/oldpage/epimeld/index_a.html).

In the present study, of the 10 clonal complexes recovered, all have previously been described in other countries, including 3 of the 43 worldwide spread resistant pneumococcal strains currently accepted by PMEN, Spain 9 V-3 (ST156), England 14–9 (ST9) and Spain 23F-1 (ST81). Spain23F-1 (ST81) and Spain9V-3 (ST156) have been present in Poland since the second half of the 1990s [2, 42]. A significant decrease in Spain23F-1 prevalence in some regions [43, 44] has been observed since the introduction of a seven-valent pneumococcal conjugate vaccine (PCV7) in 2001. Also in Poland the decrease of the Spain23F-1 clone occurrence from 19.1 to 6.8% among invasive and non-invasive PNSSP isolates was noticed [2, 42]. In our study, this clone constituted 10% of PNSSP isolates. Spain23F-1 is considered as a clone with low deposition of causing invasive diseases, and its adaptation to persistent colonization of the human nasopharynx may facilitate its intercontinental distribution [45]. The spreading of two related clones Spain9V-3 (ST156) and ST143, and their representatives seems to be responsible for the increase in resistance observed in 2002 in Poland [42]. Between 2002 and 2005, the Spain9V-3 complex spread in Poland from 22 to 47.5% of PNSSP isolates [2, 42]. In our study, CC1 (ST156/ST143/ST10340) isolates represented 30% of PNSSP isolates. Penicillin nonsusceptible variants of England14–9 (ST9) for the first time have been reported in Poland in 2004–2005 years [2] and in our study this clone with variants represented 20% of tested pneumococcal strains. Some of the previously detected clones [19] were found again, including ST87, ST135 and ST320 strains.

## Conclusions

The high persistent prevalence of antibiotic-resistant *S. pneumoniae* strains in children with AOM after the completion of the therapy seems to confirm that the unsuccessful bacterial eradication may be regarded as a risk factor of infection recurrence. Presence of PCV serotypes as well as PMEN resistant clones in tested children are confirmation of their spreading caused by minimal uptake of PCVs in Polish population.

## Additional file

Additional file 1: Analysis of the similarity among tested isolates and PMEN strains made by eBURSTv3 software. (DOCX 56 kb)

## Abbreviations

AOM: Acute Otitis Media
CC: Clonal Compex
CI: Confidence Intervals
CRP: C-Reactive protein
DCC: Day care center
ENT: Ear-Nose-and-Throat
EUCAST: European Committee on Antimicrobial Susceptibility Testing
Hib: Haemophilus influenzae type b
MEF: Middle Ear Fluid
MLST: Multilocus Sequence Typing
N: New case AOM
NP: Nasopharyngeal
OP: Oropharyngeal
OR: Odds Ratio
PMEN: Pneumococcal Molecular Epidemiology Network
PNSSP: Penicillin Non-Susceptible S. pneumoniae
PSSP: Penicillin Susceptible S. pneumoniae
R: Recurrent AOM
RR: Relative risk
Sp: Streptococcus pneumoniae
ST: Sequence Type
TF: AOM treatment failure
WBC: White blood cells
