# Supplementary material for: Resistant Streptococcus pneumoniae strains in children with acute otitis media– high risk of persistent colonization after treatment
Source: BMC Infect Dis. 2018 Sep 25;18:478. doi: 10.1186/s12879-018-3398-9 (PMC6156860; doi:10.1186/s12879-018-3398-9)
Supplement: Supplementary file 1 — Analysis of the similarity among tested isolates and PMEN strains made by eBURSTv3 software. (DOCX 56 kb) [file 12879_2018_3398_MOESM1_ESM.docx]

**Analysis of the similarity among tested isolates and PMEN strains (highlighted) made by eBURSTv3 software**

eBURST Report - Mon Sep 12 13:45:31 CEST 2016

No. isolates = 68 | No. STs = 57 | No. re-samplings for bootstrapping = 1000

No. loci per isolate = 7 | No. identical loci for group def = 6 | No. groups = 6

Group 1: No. Isolates = 6 | No. STs = 3 | Predicted Founder = 15

Average ST Bootstrap

ST FREQ SLV DLV TLV SAT Distance Group Subgrp

15 2 2 0 0 0 1.0 34% 0%

423 2 1 1 0 0 1.5 0% 0%

9 2 1 1 0 0 1.5 0% 0%

Group 2: No. Isolates = 3 | No. STs = 2 | Predicted Founder = None

ST FREQ SLV DLV TLV SAT Distance

87 2 1 0 0 0 1.0

10342 1 1 0 0 0 1.0

Group 3: No. Isolates = 3 | No. STs = 2 | Predicted Founder = None

ST FREQ SLV DLV TLV SAT Distance

81 2 1 0 0 0 1.0

2033 1 1 0 0 0 1.0

Group 4: No. Isolates = 3 | No. STs = 2 | Predicted Founder = None

ST FREQ SLV DLV TLV SAT Distance

320 2 1 0 0 0 1.0

9062 1 1 0 0 0 1.0

Group 5: No. Isolates = 2 | No. STs = 2 | Predicted Founder = None

ST FREQ SLV DLV TLV SAT Distance

374 1 1 0 0 0 1.0

63 1 1 0 0 0 1.0

Group 6: No. Isolates = 3 | No. STs = 2 | Predicted Founder = None

ST FREQ SLV DLV TLV SAT Distance

143 2 1 0 0 0 1.0

10340 1 1 0 0 0 1.0

Singletons: size 44

230

199

135

53

20

193

615

327

191

384

289

448

18

156

124

75

185

41

315

180

377

376

344

218

217

505

242

273

270

37

113

177

67

433

496

175

173

338

306

304

90

205

268

236

**Figure S1 Diagram MLST - comparison of tested strains and 43 PMEN strains.**


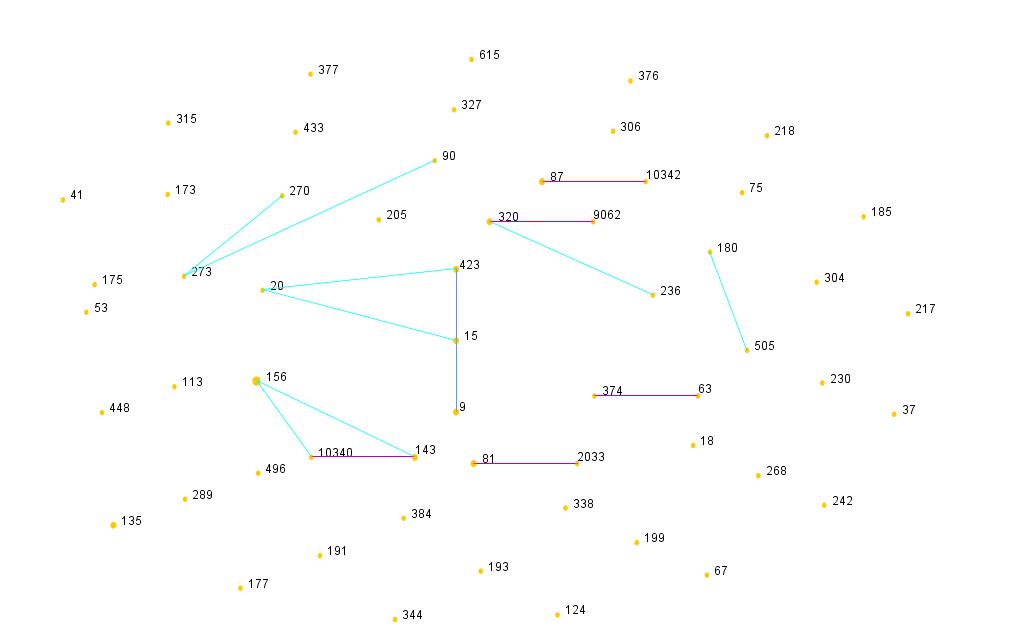


**The size of the circle (symbolizing STs) corresponds to the number of isolates belonging to a ST. Single-locus variant and double-locus variant links are represented by pink and blue lines, respectively.**
